# Quality Challenges in Municipal Telecare Call Center Services: Qualitative Evaluation Using the Anchored, Realistic, Cocreated, Human, Integrated, and Evaluated (ARCHIE) Framework

**DOI:** 10.2196/76054

**Published:** 2026-03-10

**Authors:** Linda C Grøndal-Eeles, Janne Dugstad, Hilde Eide, Etty Nilsen

**Affiliations:** 1 Centre for Health and Technology, Faculty of Health and Social Science, University of South-Eastern Norway, Postboks 4, Borre, 3199, Norway, 47 95109280

**Keywords:** quality, municipalities, telecare services, telecare call center, assistive technology, patient safety, telecare, call center

## Abstract

**Background:**

Telecare is seen as a promising technology aimed at enhancing the accessibility and efficiency of health care services. Although focus on quality has been highly prioritized within the health care services, there is a need to explore the quality of telecare services in general and municipal telecare call centers (CCs) in particular, as health and assistive technologies are increasingly being implemented in patients’ homes.

**Objective:**

The study sought to explore which factors influence the quality of telecare services provided by municipal telecare CCs in Norway, evaluated through the anchored, realistic, cocreated, human, integrated, and evaluated (ARCHIE) framework.

**Methods:**

The study had a multiple-case design. Interviews were the main source of data from 15 informants from 5 municipal telecare CCs across Norway. Observation and document studies were used for background and contextualization. To explore and evaluate quality, a combined deductive–inductive analysis was conducted.

**Results:**

Evaluated against the ARCHIE framework, none of the quality criteria were fully met. Due to the telecare service not being sufficiently anchored for all patients, it was challenging to provide realistic technologies. The collaborative work was difficult, with challenges in recruiting patients. The human principle was characterized by variation of knowledge and national guidelines. Municipal telecare CCs were not integrated into the health care services, and data must be used to a greater extent for evaluation and learning than is currently the case.

**Conclusions:**

The findings suggest that municipal telecare CC services have several shortcomings in providing high-quality health care. Relating the quality principles identified by the ARCHIE framework to normalization process theory constructs indicates that the CC service remains in a transitional phase of normalization. To improve the telecare CC services and enhance communication and integration, policymakers need to reduce fragmentation in the broader health care system. Further national standardization to professionalize the telecare CC services should be developed. The telecare CCs need to improve their service related to all indicators of the ARCHIE framework. Training for telecare operators should be prioritized.

## Introduction

Telecare and digital technologies have been partly integrated into the health care services of Western countries during the past decade. Telecare can be defined as: “the use of information, communication, and monitoring technologies which allows health care providers to remotely evaluate health status, give educational intervention, or deliver health and social care to patients in their homes” [1]. A call center (CC) is a 24/7 service that operates telecare. A CC employs operators, typically health care workers or technicians, to answer, document, and assess calls and alarms from assistive technology [2]. Assistive technology is an umbrella term for assistive products and their related systems and services, which help maintain or improve an individual’s functioning related to cognition, communication, hearing, mobility, self-care, and vision [3,4]. These technologies are implemented in people’s homes and are the reason for the CCs’ existence. CCs organize their service in a variety of ways, including their core service, which is to answer, evaluate, and document assistive alarms and take any necessary further action [2]. They also provide additional services, including ambulant teams, advisory departments, or monitoring technologies that are not health-related. The CCs may be private entities or part of the public service. In Norway, the CCs are largely organized as intermunicipal collaborations that are hosted by one municipality and regulated by the Local Government Act [5]. Our recent article investigating the organization of municipal telecare CCs in Norway found that the centers were in an early development phase and had different organizational models, leading to a variation of delivered services [6]. As CCs are considered new and innovative within the health care service and provide the service in a new way, there is a need to investigate the quality of their services.

The World Health Organization (WHO) defines quality as “the degree to which health services for individuals and populations increase the likelihood of desired health outcomes and are consistent with current professional knowledge” [7]. To achieve high quality within health care services, several factors, such as patient safety, effectiveness, availability, and patient-centered care, need to be evaluated [8], and many governments have strengthened their focus on quality within the health care system [9-12]. International studies have shown a multitude of adverse events happening within the health care system each year, which have an effect on quality [13-16].

Several frameworks have been developed for the evaluation of the quality of health care services. One of the earliest and most used is the Donabedian framework [17], which evaluates the systems, processes, and outcomes of health care delivery. The framework has also been used for evaluating quality in digital services such as telecare [18]. Tossaint-Schoenmakers et al’s [18] review highlights 3 broad aspects that are important for integration: the needs of the care recipient, technology that is attuned to the organization and care processes, and human resources that are aligned with the desired end results. A need for more in-depth studies into the specific processes in different parts of the health care system related to eHealth is suggested.

Quality in telecare has increasingly become a relevant topic, as more assistive technology is being implemented. The aim of implementing technology is to improve access to the health care system and improve effectiveness [19]. The use of telecare can improve communication between health care professionals as well as improve patient safety by reducing errors [20,21]. Furthermore, digital health care can give patients the opportunity to participate in their own treatment and treatment plans to a higher degree than before [19]. Challenges and barriers related to the use of telecare include technical barriers, such as low internet access, poor digital competence among certain user groups, and concerns about privacy and data security [22]. In addition, Woolham et al [23] mention cost-effectiveness, assessment and deployment, ethical implications, and user involvement as key challenges.

As there is a need to explore in greater depth how quality is performed [18], Greenhalgh et al’s [24] framework, called the anchored, realistic, cocreated, human, integrated, and evaluated (ARCHIE) framework, seems to be a relevant framework to evaluate important quality aspects of telecare. The framework determines whether a service is anchored, realistic, co-creative, human, integrated, and evaluated, constituting the acronym ARCHIE. The framework was based on interviews and observations in various care contexts to identify barriers and suggest several solutions to provide quality care with useful assistive technology. An important takeaway from Greenhalgh et al’s [24] study is that technological advances should be underpinned by a user-centered approach to design and delivery. The care contracts and technology should be personalized for each patient, from branded products to interoperable components. Furthermore, the technology should have the ability to be used flexibly across devices and platforms [24].

The benefits of telecare are still not well-established [25,26]. If the service lacks quality, this can lead to an increased burden of illness, increased economic costs, and medical errors such as inaccurate diagnoses, medication errors, and inappropriate treatments [27,28]. If clinical guidelines are not followed, this can result in ineffective care. By increasing quality within the telecare service, the benefits of the implemented technology might be easier to recognize.

Patient safety in primary care is a critical aspect of health care. According to the WHO, patient safety involves the prevention of errors and adverse effects associated with health care, aiming to “do no harm” to patients [29]. Research indicates that incidents in primary care, although often less severe than those in hospitals, significantly impact overall patient safety due to the high volume of patient interactions [14]. Furthermore, it is important to take into consideration that patients will increasingly receive complicated treatment of complex illnesses at home. As previous research has highlighted, the assessment of quality and safety culture is a fundamental step in understanding health care providers’ safety-related perceptions [14,30]. Therefore, fostering a robust safety culture and implementing targeted interventions for quality are paramount in primary care.

Telecare has the ability to enhance the accessibility and efficiency of health care services [31]. However, a systematic review identified multiple patient safety risks associated with telecare, including issues related to the nature of homecare tasks, person-centered characteristics, technology and devices, organization and environmental factors [32]. These risks can arise from the complexity of integrating technology into home care settings, where both the physical environment and the patient’s capabilities play crucial roles. A qualitative study highlighted the importance of care workers’ roles in ensuring patient safety through telecare [33]. The study identified 3 essential work processes: aligning people and technologies, being alert and staying calm, and coordinating activities based on people and technology [33]. These processes underscore the need for effective training and support for care workers to manage the interface between technology and patient care effectively.

Few studies have investigated telecare providers’ perspectives on how quality is performed in municipal telecare CC practice. Therefore, the aim of the study was to explore which factors influence the quality of telecare services provided by municipal telecare CCs in Norway, evaluated through the ARCHIE framework.

## Methods

### Study Design

This study had a multiple-case design. A multiple case study is regarded by many as more robust than the single-case design [34-37]. In this study, similar cases have been studied in order to gain a broad understanding of quality, and not to compare the individual cases. The various cases form a bricolage [38] that embraces the complexity of the context. Interviews were the main source of data. Data from observations and documents have been used to enhance the understanding of the background and context of the CCs.

### Recruitment and Data Collection

Data were collected in May and June of 2022. At the time of data collection, no overview of Norwegian telecare CCs existed, so a snowball-inspired strategy was used in our networks to identify centers. An email was sent to the managers of 5 CCs, and they agreed to participate with their leaders and personnel.

A semistructured interview guide was developed for the in-depth interviews. The study’s aim was used to develop themes for the interview guide where the following themes were covered: (1) The participants’ understanding of the municipal telecare CC service in their context, (2) How the municipal telecare CC services operated, (3) Cooperation with other health care services, (4) Ethical challenges, (5) Competence requirements and competence development, and (6) Perspectives on the further development of the municipal telecare CC services, to shed light on the aim. The idea was to let the informants speak as freely as possible within the themes. One paper has previously been published based on the same in-depth interviews, focusing on the organization of the services [11]. For this paper, the interviews were analyzed focusing on quality.

The first author, LGE, visited 4 of the CCs, with each visit lasting for one day, including interviews, a tour, and observations. On arrival at the CCs, a guided tour was conducted. The observations included how the CC operators worked, how they responded to alarms, the layout of the facilities, the workstation setup, and some of the technology included in the services (eg, social alarms, GPS, medicine dispensers, door locks, and sensors). These observations provided important background knowledge for understanding the service and supporting the interviews. Notes were taken and used to understand the context.

All the interviews were performed by LGE following the semistructured interview guide, which constituted the main source of data for the studies. As the interview guide contained broad, open questions, the informants could speak as freely as they wanted. Four of the 5 CCs were visited, while one CC had to be interviewed digitally due to the COVID-19 pandemic restrictions. Ten informants were interviewed face-to-face. Two of these were conducted with 2 informants present, and the rest were conducted one-on-one. Five informants were interviewed digitally—one with 3 informants present, and 2 were conducted one-on-one. The informants were present at the CCs during the interviews, and this was also the case during the digital interviews.

The interviews lasted from 45 minutes to 2 hours and were audio-recorded and transcribed.

### Data Analysis

The data analysis had a combined deductive–inductive approach, analyzing the CCs as the unit of analysis. First, text segments were identified and sorted in accordance with the specific principles in the ARCHIE framework in a deductive fashion. Second, the text under each principle was analyzed inductively.

### Deductive Analysis Using the ARCHIE Framework

The ARCHIE framework was designed to be adaptable and to guide the development and evaluation of telehealth and telecare services in various contexts [24]. The framework aims to operationalize quality in telehealth and telecare in order to improve the proportion of patients receiving appropriate, acceptable, and workable technologies and services and has 6 principles (Table 1).

**Table 1.** Overview of the ARCHIE (Anchored, Realistic, Cocreated, Human, Integrated, and Evaluated) framework [24].

Principle	Content
Anchored	Having a shared understanding of what matters to the patient, which includes design and development of technology
Realistic	Understanding the natural history of illness. The illness may progress, and the patients’ needs change
Co-creative	Evolving and adapting solutions with patients. This should be continuous rather than standardized
Human	Supporting patients through interpersonal relationships and social networks where knowledge and information are key
Integrated	Developing mutual awareness and sharing knowledge
Evaluated	Ensuring system learning from experiences built over years. Ongoing innovation to increase quality

- Step 1: The first author (LGE) transcribed the 11 interviews and initially read through all of them, identifying and coding text segments that aligned with the 6 main ARCHIE principles described above. Some text segments aligned with the ideal quality principle, but others were inconsistent; these inconsistent statements were coded under the specific principle as “not” (Table 2 contains examples). Consequently, all the coded text had a main code in accordance with ARCHIE, and if relevant, a subcode as “not.”

**Table 2.** Examples of coding that align with the ARCHIE (Anchored, Realistic, Cocreated, Human, Integrated, and Evaluated) principles and which are inconsistent with the ARCHIE principles.

Principle	Example
Anchored	A shared understanding of the needs of the patient
Not anchored	A patient is given the wrong technology
Realistic	Understanding the natural history of illness
Not realistic	Change of pattern for the use of assistive technology, but the patient still has the technology

- Step 2: All authors read and coded 3 selected interviews to align the understanding of the ARCHIE framework principles and secure the initial coding in each principle. The tentative coding by the first author in Step 1 was discussed, adjusted, and agreed on.
- Step 3: LGE reread and checked the coding of all the interviews.

### Inductive Analysis

After the deductive analysis, a second phase followed using inductive content analysis within each ARCHIE principle. The inductive analysis followed the structured process described by Elo and Kyngäs [39], with 3 main steps: preparation, organization, and reporting.

- Step 4: In the preparation phase, the text within each ARCHIE principle was re-examined by LGE to identify meaning relevant to the principle’s theme. These units were extracted and organized for a deeper analysis.
- Step 5: During the organization phase, the author group’s focus was on identifying patterns, contrasts, and recurring themes within each principle, rather than generating new codes. For example, in the anchored principle, both aligned and misaligned statements were examined to understand how services reflected, or failed to reflect, what mattered to the patient. This was discussed in the author group to ensure understanding of each principle.
- Step 6: The reporting phase synthesized insights from each principle to highlight trends and tensions. These were then interpreted in relation to the ARCHIE framework to assess whether and how quality was met across the principles. NVivo (version 5.0; Lumivero) was used in the coding processes.

### Rigor and Trustworthiness

To ensure rigor and trustworthiness in the study, criteria developed by Guba and Lincoln [40] were followed, including credibility, transferability, dependability, and confirmability. To ensure the credibility of the coding, analysis, and results were discussed in several rounds, in the deductive as well as the inductive phase of the study, to ensure the group of authors could express their thoughts, concerns, or other factors. One issue that the author group discussed in several rounds was how to interpret the content of the principles in ARCHIE, and which statements belonged where. To understand the principles, the author group went through each statement, forming a consensus on whether or not statements were placed correctly in our understanding of what each principle should contain.

The study provided detailed descriptions of the sample to ensure transferability and support contextual understanding. The coding process was well-documented and logged in NVivo, as well as revisions to maintain dependability. In any research, the expulsion of all biases is challenging. However, the research group consisted of authors with interdisciplinary backgrounds, such as nursing (LGE and HE), economics and management (EN), optometry (JD), psychology, and eHealth communication and counseling (HE). The group also had broad competence in research methodology as well as health technology implementation. This meant that the authors could share different views on the research and ask critical questions to make sure the author group’s assumptions and interpretations were as transparent as possible, thereby ensuring confirmability. Furthermore, the coding process was repetitive and reflexive, guided by Saldaña’s [41] approach, and involved multiple rounds of discussions within the author group to reach consensus. Analytical transparency was maintained through NVivo documentation.

### Ethical Considerations

The study was approved by the Norwegian Agency for Shared Services in Education and Research, which assessed the data processing plan to be in accordance with data protection legislation (Ethics approval number: 426173). Furthermore, the main principles for research according to research ethics in the social sciences and the humanities [42] were followed. All informants received written and oral information about the study, and informed consent to participate was obtained from each informant. The informants participated voluntarily and received no compensation of any sort. The CC informants appreciated that researchers took an interest in the telecare topic and therefore welcomed the study. Data were stored and secured in a USN-protected area. The data in the quotes used throughout this article were anonymized.

## Results

### Overview

The first section of the results will present the sample of the study, and key dimensions of quality according to the ARCHIE framework will then be presented. The headings represent the main findings in each principle.

### Sample

Data were drawn from 5 municipal telecare CCs in different parts of Norway (Table 3). Eleven interviews were performed with 15 professionals working in the 5 CCs (CC1-CC5). Five informants from CC1 were interviewed, 1 from CC2, 4 from CC3, 2 from CC4, and 3 from CC5. In total, 12 women and 3 men were included. The informants had a variety of professional backgrounds and roles within the centers: 6 were managers, 4 were advisors, 4 were health care operators, and 1 was an IT technician.

**Table 3.** Overview of call centers (CCs) participating in the study.

	CC1	CC2	CC3	CC4	CC5
Number of municipalities in cooperation	14	16	16	6	12
Number of citizens in the cooperating municipalities	Estimated 140,000	Estimated 175,000 (Estimated by authors)^a^	Estimated 306,000 (Estimated by authors)^a^	Estimated 51,000	Estimated 85,000
Established	2019	2003 serving fire and rescue. Included in the health care service from 2019	2018	2019	2016 under fire and rescue service, included in health care 2023, colocated with municipal accident and emergency department.
Organization (departments or services provided alongside telecare CC)	Colocated with municipal accident and emergency department	Monitoring technical alarms in public buildings (fire alarms, lift alarms, etc)	Ambulatory team department. (Can visit patients whose alarms cannot be resolved by phone)	Service design advisory department. (Can assist municipalities in cooperation with implementation strategy and other eHealth challenges	Monitoring technical alarms in public buildings (fire alarms, lift alarms, etc)

a Estimated by authors.

### The Unanchored Services of Call Centers

The anchored principle was meant to reflect a shared understanding of the patient’s needs between the patient and all the stakeholders involved in the provision of the service, emphasizing what is important and meaningful to the patient. However, the allocation of assistive technology did not appear to be anchored with all the stakeholders working with each patient. The telecare providers working in the CCs were not involved in the process of assessing the patients’ needs or prioritizing and selecting what services they should receive. Informant K said:

*The assumption must be that the home nursing staff, in collaboration with other decision-makers in the municipalities, should work much closer with the patient to see if the technology can actually relieve the burden. It is felt that this is not being done.*

**Figure 1.**
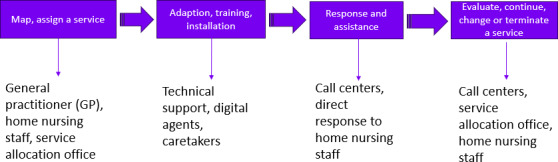
Overview of the actions related to the telecare call center service and stakeholders within primary health telecare.

The municipal telecare CCs provided services across several municipalities, which otherwise worked independently of each other. In order to establish and provide the service for a patient over time, the telecare services included several stakeholders within the health care services of each municipality, who interacted with the patient during the course of the service. Initially, the home nursing unit and the service allocation office were involved in the referral and mapping of the patients and the assignment of the service. In addition, general practitioners, family members, and even the patients themselves could identify the need for a service in the early phases. When a patient was allocated a telecare service, the health technology support team was responsible for installing the technological devices in the patient’s home and instructing the patient in the use of the technology. The CC was then notified. Their responsibility was to respond to alarms and calls and to monitor the technology and incoming data. In their daily operations, the CC alerted the home nursing staff if physical interaction with the patients was needed. In order to change the service for a patient, the CCs or home nursing staff reported their concern to the service allocation office. A suspicion that the service was perhaps no longer what the patient needed would prompt an evaluation and the allocation of new services. Figure 1 displays a visualization of the process with an overview of the relevant stakeholders involved in the different phases of assigning and providing a telecare service.

### The Challenge of Having Realistic Technology When the Needs Are Constantly Changing

The realistic principle meant that technology and care packages needed to allow for the patients’ conditions to progress or change, thereby supporting changes in the patients’ needs. However, if the technology was not anchored in the first place, it could never be realistic. The informants addressed how patients received the technology too late, where the situation of the patients was rapidly changing, and the technology was therefore not realistic. Informant K explained:

*We feel like the introduction to health technology should happen at a much earlier stage than what is being done now.*

Another challenge was how some progressive illnesses left the patient in a state where they did not know, understand, or remember the different assistive technologies, indicating that their health situation had changed, so they were no longer capable of using the technology as intended. When a patient reached this stage, there was no benefit for either the patient or the health care service, because the assistive technology was a poor fit. As informant F stated:

*When a patient has moderate Alzheimer’s, then it is too late. They will wonder what it is and maybe even take it off and not remember to use the alarm when needed.*

The CCs could pick up on the progression of a patient’s health conditions by monitoring patterns in their behavior, such as a sudden increase in calls from a patient via their social alarm. The CC reported these changes to the home nursing staff or service allocation office. Informant B said:

*We have two patients that trigger their alarms hundreds of times. Then we can say, this user has the wrong service.*

However, the CCs found that in spite of their reports, the patients continued to have assistive technology that did not work for them. This indicated that the municipalities were unsuccessful in re-evaluating whether a patient had the right technology at the right time, and also that the feedback from the CC was not taken into consideration.

### Challenges in Co-Creative and Collaborative Work

The co-creative principle meant that the technology should be continuously cocreated between the service stakeholders and the patients, making it tailor-made to a certain degree. Co-creation and interaction across disciplines and levels were raised as challenges by several informants. Lack of cooperation and the sharing of information and knowledge between CCs and other institutions was a challenge in general, and specifically on a user-related level. Informant F said:

*We see a need for cooperation, on a larger scale, with GPs and hospitals. But we also see a great need to interact better with the patients to be able to give them a better service.*

The focus of the CC providers was to cooperate and interact with patients. However, informants explained how it was a challenge to recruit patients to cooperate and test the technology. The CCs tested all the technology, and some even created a test lab where patients, future patients, and their next of kin could come and gain hands-on experience of the technologies. Informant K said:

*We have emphasised a holistic focus. We see it as a core value. Because it is about bringing people with us the whole way, from development to implementation.*

None of the telecare providers involved in this study actually developed health technology. They primarily managed calls/alarms from technology purchased by the municipalities. The technologies had been purchased based on the needs of the patients in each municipality. However, some of the CCs provided recommendations or came up with suggestions to the municipalities about what technologies to procure. In some instances, they even procured technology on behalf of the municipalities. However, it was explained that patients’ needs determined what was to be procured, not the technology in itself. Informant K explained:

*We must understand the needs to solve a problem, then we can go out and see – is there any technology that can work?*

Telecare providers had a unique insight into how the home nursing staff coped with the technology, whether they managed the technology, and whether the patients could handle the technology. Informant B said:

*We get the home nursing perspective, on what is difficult technology. Because it can be completely different for an engineer who has developed it and it makes sense to them, but maybe not to an assistant in the home nursing team.*

### The Importance of Knowledge for Nurses

The human principle comprised the interaction between patients and employees’ knowledge and key information. One CC received 300,000 alarms in 2021, with 861 events in 24 hours and 36 per hour, which underlined how much interaction the CC service had with its patients. The telecare providers frequently talked to the patients and got to know many of them. Having information and personal knowledge about some of their patients led to more calls being resolved by the CC service—hence, the alarm did not need to be escalated to the home nursing team. Informant P stated:

*Because in X [a private call centre] they just sent the call through to home nursing, but here [municipal call centre] they answer, and ask questions. “I have lost something under the sofa.” A call like this will not go out to the home care team.*

As a result of the high number of alarms, an ethical dilemma emerged regarding frequent callers. The patients labeled as frequent callers could lead to employees in the CC service feeling that they were getting tired of the patients and, therefore, needed to work to ensure that they upheld their standards and the quality of their communication. The telecare providers said that they managed it because they knew that these patients’ needs were not being met. Some reflected that the reason for the frequent calls might be the fact that the patient had the wrong technology. Informant D said:

*I have experienced that I get tired of alarms, especially when the alarm is triggered for “nothing.”*

Findings highlighted a variety of health personnel working in the CCs. Some of the CCs had nurses, some had health care workers only, and some had technicians working together with nurses as operators in the CC. Some CCs had a standard minimum requirement for at least one nurse, whereas others, which only had nurses, were looking to hire health care workers. Informant P said:

*We have made it a requirement that a nurse must be present at work at all times*.

Not only did the professional backgrounds of the operators vary, but their level of experience also seemed to differ. One of the CCs was colocated with an accident and emergency department. The nurses had a high level of competence in handling telecare and solving acute matters over the phone. Informant A said:

*We only have nurses with very high competence answering acute calls. Because that happens every week, and they need to be able to handle it.*

Employees’ experience seemed to be key. The interviews and observations revealed that there were no national decision-making tools, procedures for the receipt of alarms, questionnaires, or standards. One nurse (Informant C) working for a CC colocated with an accident and emergency department said:

*At the accident and emergency department we use triage systems [decision-making tools], but at the call centre we have nothing. There are no procedures as to what we should do.*

Where there were no decision-making tools or procedures for how to receive and handle a call, it could elevate the risk of misunderstandings, as there was no “safety net” to ensure the operators had asked about everything that was relevant when handling an alarm. One informant explained an episode where the operator did not inquire thoroughly enough during an alarm that ended with a patient lying on the floor for a long time, while the operator thought that the patient was lying in bed. Another CC, which monitored a wide variety of technology-based services, including lift alarms, fire alarms, burglary alarms, and an after-hours answering service for the municipality, needed other skills for non-health-related alarms. Informant K explained:

*We have two operators on duty – one with a health background and one with a technical background. This composition is because of the portfolio of services we have.*

However, all alarms had the same ringtone in all the CCs. In the CC where both nurses and technicians were working alongside each other, they answered alarms without any system or pattern as to which one of them was answering which alarm. The lack of alarm identification meant that the technicians answered medical alarms, and vice versa. They would assist each other if one of them, for example, answered a cardiac arrest. Informant C stated:

*If a fire alarm has gone off in an apartment, we should act right away, but if an alarm box has a low battery, we have 12 hours. But we can’t tell the difference between the two when the alarm goes off [in the call centre].*

The study found no evidence to show that there were more adverse events in CCs with health care workers than with experienced nurses. This is because there was no indication of any system for registering adverse events, or opportunities to look into whether CCs with different professional employee profiles had more adverse events than others.

### Challenges With Partly Integrated Technology

The integrated principle meant that both technology and work processes should be integrated with existing systems. Even though the CCs reported that it was demanding to cope with all the technical integrations, it seemed that this was a very high priority for all of them. They also emphasized the importance of the integrations. Informant A explained:

*Because of the integrations, we can clarify many alarms that come in*.

All the CCs had patients’ medical records integrated. This meant that the telecare providers had access to information about the patients, such as when the home nursing team visited the patients. When operators answered alarms, a notification came up on the screens, as well as an alarm or ringtone in the CC. The operator could see who was contacting them, and their medical record came up on their screens. Furthermore, it seemed that the technology used by the patients was integrated into the platforms of the CCs. However, the CCs explained how it could take time and be challenging to integrate new technology into the platforms. The CCs had many vendors to relate to and many technologies that needed to be integrated into their systems. On top of that, new technologies were making their way into the service, which also required integration.

As for collaboration and the integration of work processes, the CCs seemed to closely cooperate with the home nursing units in the different municipalities where they provided services. However, the analysis indicated an “us/them” attitude between the CC providers versus the more administrative service allocation office in the different municipalities, suggesting a lack of cooperation between the organizational units involved. Informant K said:

*I don’t know what to say about the administration – it is not that they are falling behind, but the cooperation, it has not worked so well.*

### Better Use of Data for Evaluation and Learning

The evaluated principle indicated learning from experience and improving systems accordingly. It should involve an ongoing effort to increase quality. The CCs had a lot of data from their operations, and the municipalities received reports from the CCs regarding this data on a regular basis. This data provided key information about the CC service and the patients and could be the basis for evaluation, learning, and development of the service. However, the CCs were not sure whether the municipalities used the data for learning purposes or if they used the data at all. The CCs monitored the number of alarms related to each of the collaborating municipalities and variations over the year and could provide data for individual patients, mapping whether the service was the right one for them. Informant B explained:

*Creating value through better use of data is important for the municipalities … How many ‘false alarms’ did the home nursing have to take on before the call centre? When the alarm is sent now, it is real.*

The CCs were reporting to the municipalities because they were trying to see the effects of their work, making sure they procured the right technology, and getting it documented. Informant K explained:

*We are reporting, we do it because we want to see the effects of the measures we implement. A call centre is not a quick fix, but thorough work from top to bottom or bottom to top.*

Furthermore, the CCs were evaluating if there were other services they could operate to make them more resilient. Many options were considered at the idea stage, but digital home monitoring was a trend that many of the CCs were looking at implementing as a service. Some CCs had already started. Informant L said:

*Digital home monitoring and rehabilitation. We are investigating the possibilities*.

## Discussion

### Principal Findings

Using the ARCHIE framework to evaluate quality in municipal CC services reveals that, despite efforts, the service remains fragmented and therefore poses a potential threat to quality. The findings in this study appear not to reflect the quality principles in the ARCHIE framework and what is indicated as high-quality telecare. The framework promotes patient-centered care, whereas this study suggests that the patients’ voices are barely heard, and it appears that consideration of what matters to the patients is lacking. This permeates throughout the following principles of the ARCHIE framework: anchored, realistic, cocreated, and poses challenges to the human principle.

In the following, the results will be discussed using the normalization process theory (NPT) [43]. NPT is a framework or set of tools concerned with understanding 3 main issues: implementation, embedding, and integration of new or modified practices, and these align closely with the challenges identified in this study. Using the ARCHIE principles, the study has researched the quality of municipal telecare CCs on a micro level [44]. To further specify how municipal telecare CCs can become part of normal digital service delivery on a meso level [44], NPT offers valuable insight and aids the assessment of how far municipal telecare CCs have come in terms of integration into routine practice, and what is missing to become a fully embedded, high-quality digital health service.

### Lack of Quality Challenges Telecare Call Center Normalization

#### Overview

In this study, the ARCHIE framework [25] was chosen because it was specifically developed as a set of quality indicators for telecare services. ARCHIE emphasizes values such as person-centeredness, coproduction, and inclusivity, which are particularly relevant in the context of digital health and remote care [25]. ARCHIE provides a useful framework for assessing how telecare services align with the intended values of care delivery. Its design makes it especially suitable for evaluating the quality dimensions of telecare implementation, which appear to be less visible in more structural models such as Donabedian’s [18,24]. Although foundational in health care quality assessment, the Donabedian model [18] focuses primarily on structure, process, and outcome and may not sufficiently capture the relational and participatory aspects of telecare services.

Of particular relevance in the NPT framework are the mechanisms that inhibit or promote the new practice, namely the following: (1) coherence, (2) cognitive participation, (3) collective action, and (4) reflexive monitoring [45]. In the following, each principle will be briefly presented. The results from the analysis of the relevant ARCHIE principles will then be discussed, including implications.

**Figure 2.**
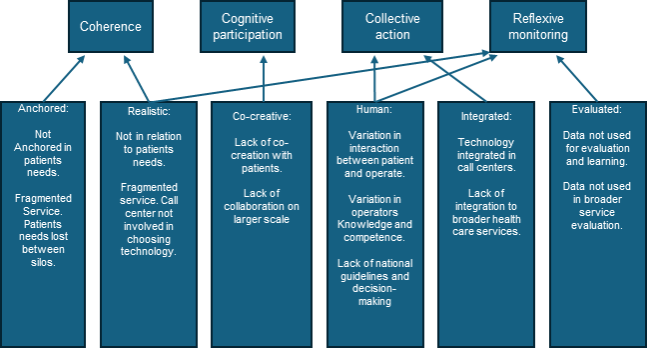
Results of the ARCHIE (Anchored, Realistic, Cocreated, Human, Integrated, and Evaluated) analysis on the call center level related to a broader implementation framework, normalization process theory (NPT), at the meso level.

Figure 2 illustrates how the principles from the ARCHIE framework align with the NPT mechanisms and findings of this study.

#### Coherence: Lack of Shared Understanding

On a meso level, the NPT construct Coherence is the process, sensemaking, and work to understand how a technology or service is implemented into practice, and also the routine for embedding a practice [45]. Coherence is regarded as a cognitive construct [43]. On a micro level, the ARCHIE principles anchored and realistic relate to coherence. Anchored links to the patient and to a shared understanding of the patient’s needs, which correlates well with coherence. Realistic looks to technology, and whether the technology is realistic for the patient [24]. Although the realistic principle may relate to several NPT constructs, an intervention that is not meaningful will rarely be experienced as realistic. In the ARCHIE principle, realistic was linked to coherence.

Findings in this study reveal a lack of coherence in municipal telecare CC services, particularly in terms of a lack of shared understanding of the patients’ needs. This could result in patients being assigned inappropriate technology. The lack of coherence becomes visible when seen through the principles of the ARCHIE framework and suggests that it is a system feature where one principle affects the other. If the decision by the service allocation office is not anchored, this will have a domino effect on the following principles, and they will not be right either. This shows how the telecare service is fragmented, and several silos are unsuccessful in working together to execute the service.

Dillon et al [46] found that when the health care system is perceived as fragmented, the outcome could be a loss of trust and difficulties navigating between providers. These results may be transferable to the telecare service. Furthermore, fragmentation may substantiate the “us/them” feeling, causing an even greater division between the stakeholders, as this study implies. Dillon et al [46] recommend a fundamental restructuring of the health care system to coordinate patient-centered care to meet the needs of the patients and to rebuild trust in the health care system. The ARCHIE framework also promotes patient-centered care, but results from this study show that the lack of tailored approaches may hinder quality in telecare and in the CC services, despite efforts by municipal telecare CCs to improve and professionalize their services.

#### Cognitive Participation: Uneven Stakeholder Engagement

The NPT construct Cognitive participation relates to the process that needs to be undertaken by individuals to achieve engagement for the implementation of a new practice on a meso level [43]. On a micro level, cognitive participation can be seen in relation to the ARCHIE principle of co-creation. This principle was not met, and the result can be divided into two. First, the lack of collaboration on a larger scale, with other health care services, and second, the lack of collaboration and co-creation with patients. Interestingly, the informants said that it was difficult to engage the patients in co-creation. Fusco et al [47] identified low health literacy, lack of understanding, time constraints, and intimidation of the co-creation process as barriers to successful co-creation. The reasons why the municipal telecare CCs in this study found it challenging to engage patients can only be speculated on, assuming that Fusco et al’s [47] barriers also apply to municipal telecare CCs.

#### Collective Action: Fragmented Operational Routines

On a meso level, Collective action relates to the work individuals and organizations need to do to enact implementation [43]. On a micro level, the ARCHIE principles human and integrated align with collective action. The findings show that operational routines within municipal telecare CC services are fragmented, the staff receive limited training, and there is a lack of standardized protocols and integration with the broader health services. These barriers hinder the enactment of quality-enhancing practices and suggest that municipal telecare CCs have not yet been fully normalized within municipal health care structures. Although key to telecare delivery, the municipal telecare CCs seem to operate in partial isolation from other stakeholders, which further complicates coordination and continuity of care.

The other ARCHIE principle closely linked to collective action is human. This study has mapped a variety of health professions working in municipal telecare CCs, spanning from highly experienced nurses working in both emergency rooms and CCs to technicians. Given that remote care is a different way of providing care, specific skills are required [48,49]. The CCs have to be prepared to handle emergency alarms, and findings indicate that the staff’s experience level is key. Anker-Hansen and Johansen [50] found that the nurses ensure patient safety in complex remote decision-making in telecare. The complexity includes making accurate decisions based on unclear patient descriptions and challenges with communication [50]. The telecare nurses emphasized the importance of clinical expertise and the challenging nature of the decision-making processes and communication. They asserted that the provision of telecare requires that the staff working in CCs must have both proper training and experience, which is not the case in this study. Proper training and protocols can help to mitigate these potential patient safety risks [32].

Data from this study shows that miscommunication and adverse events have occurred. In Fisk et al’s [48] study, the emphasis is on the need for a diverse range of skills and knowledge, which may include a mix of health care professionals. There is a lack of national systems, updated guidelines, procedures, and best practices for handling alarms to guide the operators working in the CC, which may negatively impact the quality of the service. Hughes [48] suggests that most adverse events result from faulty systems and processes rather than individual errors. This raises concerns about the impact of nonoptimized systems in telecare services.

In 2014, Guise et al [32] called for increased standardization in reporting adverse events to improve the knowledge base in telecare, but current research indicates that little progress has been made since. Conversely, there are critical voices expressing concerns about over-reliance on standardized guidelines and protocols. It is argued that standardization can lead to a lack of flexibility and creativity in addressing complex, real-world problems, and that these guidelines sometimes stifle critical thinking and innovation, leading to a more bureaucratic and less-effective health care system [49]. The importance of reflexivity and questioning established norms is stressed, and it is argued that a one-size-fits-all approach to guidelines can overlook the unique contexts and needs of different health care settings and patients. It seems that the absence of national guidelines or standards further contributes to the disparity of collective action being varied, making it difficult to foster collective ownership of telecare quality improvement.

#### Reflexive Monitoring: Limited Evaluation and Learning

Reflexive monitoring reflects the evaluation of a new practice and the effects the new practice has. This includes assessment of the advantages and disadvantages of the new practice [45]. On a micro level, reflexive monitoring aligns with several of the ARCHIE principles: realistic, human, and evaluated. This implies that both the ARCHIE framework and NPT are closely related to practice, gliding into each other, and can be challenging to separate. The ARCHIE principles of human and realistic have been discussed under other NPT constructs; however, they are also relevant on a meso level of reflexive monitoring because they emphasize continuous assessment and adaptation based on human experience and context.

In the ARCHIE framework, the insufficient and ineffective utilization of data falls under the evaluated principle [24]. The results of the study reveal that few municipal telecare CCs have mechanisms for evaluating their performance or for incorporating feedback from patients. This lack of systematic evaluation limits opportunities for learning and adaptation and contributes to persistent gaps in quality and safety. Without reflexive monitoring, it is difficult to identify what works, what needs improving, and how services can evolve to meet patient needs more effectively. The effective utilization of data in telecare services is crucial for enhancing patients’ outcomes and service efficiency. When data is not coherently managed or used, it can lead to fragmented care, missed opportunities for service improvement, and reduced patient trust [51]. Inefficiencies such as repeated tests and procedures may also arise, increasing operational costs [52]. This concern is seen in the WHO’s SMART Guidelines [53], which emphasize that digital technologies should be designed to support consistent, standards-based data use to improve care quality and coordination.

Regular audits and feedback mechanisms are also vital for continuous improvement [54]. Furthermore, Guise and Wiig [49] encourage training that includes patient-centered methodology to ensure good patient–professional relationships in remote care.

There are several challenges regarding the telecare service that need to be addressed on a meso level: lack of coherence, effective utilization of data, and outdated national guidelines. From a system perspective, health care is seen as a complex set of management levels, along with the policies, resources, and actors involved in delivering care [55], referring to the entire network of organizations, people, and actions whose primary intent is to promote, restore, or maintain health [56].

### Strengths and Limitations

The ARCHIE framework was selected for this study because it was especially developed for defining quality in telecare. The framework has successfully assisted the research, uncovering quality and patient safety challenges within the telecare service, including municipal telecare CCs.

This study’s combined deductive–inductive approach to the ARCHIE framework contributes to an enhanced understanding of the complex nature of the municipal telecare CC service.

However, when analyzing the data, we found that the different principles in ARCHIE were difficult to differentiate from one another and could be intertangled, meaning that the data could fit several of the principles. This was resolved by being precise in choosing statements and through discussions within the author group, to ensure that the statements reflected how the group understood the principles.

The selection of the municipal telecare CCs was a potential limitation for the study. At the time of data collection, modest information was available on existing CCs, and the selection was therefore random for practical purposes. As we have learned in the process of writing this study, however, the CCs under study represent the most common way of organizing CCs in Norway. The ease of access to informants might have compromised the reliability and validity of the data [57]. Another limitation to be aware of is that of the cross-sectional snapshot, as data were collected at one point in time in this study. This may not represent long-term behaviors or trends.

The lack of national standards makes it challenging to benchmark quality, which affects both analysis and interpretation.

A strength of the research is the diverse background of the research group. This interdisciplinary background enabled the group to conduct an open and critical process, with the aim of reducing bias as much as possible.

### Conclusions

The findings of this study suggest that telecare services in Norway are not yet fully normalized. The challenges identified across all 4 NPT constructs—conceptual ambiguity, uneven engagement, fragmented routines, and limited evaluation—indicate that telecare remains in a transitional phase of implementation. The application of the ARCHIE framework [24] helps to clarify the current position of telecare within the health care system and highlights specific areas where targeted interventions could accelerate normalization. Combined with NPT, which offers critical insights into the processes necessary for change at the meso level, this study provides a dual insight for understanding both the process and the quality dimensions of telecare. These insights are critical for policymakers, service designers, and practitioners aiming to improve the quality, safety, and integration of telecare services.

### Implications for Policymaking

The findings of this study imply that municipal telecare CCs need to improve the telecare service to achieve the full potential of integrating a high level of quality and patient safety. Improvements should be considered at both meso and micro levels, as shown in this study. At a meso level, one way to advance is for stakeholders in telecare services to improve communication across their services and reduce fragmentation in the system. The telecare service calls for national standardization and the professionalization of the service. On a micro level, all of the ARCHIE principles need improvement. Training for telecare operators is one area that should be prioritized. As shown in this research, Greenhalgh et al’s [24] ARCHIE framework can be a useful tool for assessing quality in emerging services within the health care domain.

### Issues for Further Research

The following issues for further research to improve quality were identified in this study:

1. The patient perspective remains underresearched, as this study has focused on the municipal telecare CCs.
2. Cooperation and communication between stakeholders—in particular, how do the municipalities consider patients’ needs for assistive technology, and how are concerns about ill-fitted assistive technology handled?
3. Competence development—what kind of training do telecare operators receive before they start as operators, and what plans are in place to maintain and increase knowledge?

## Abbreviations

ARCHIE: anchored, realistic, cocreated, human, integrated, and evaluated
CC: call center
NPT: normalization process theory
WHO: World Health Organization
